# Metal Therapeutics

**Published:** 1885-02

**Authors:** 


Mental Therapeutics.—In the course of his address before the New York State Medical Association, Prof. Austin Flint said: "The physician who appreciates the importance of mental therapeutics, and of the duties incident thereto, will not fail to hold out to patients the encouraging features of a case. He will not give way to gratuitous forebodings. He will be circumspect in forming, and still more in announcing to his patients, an unfavorable prognosis. He will be slow to hazard a prediction as to the precise date that a disease will prove fatal, and still less will be guilty of the brutality of imitating a judicial sentence of death. He will keep out of the view of his patients discouraging possibilities, but not those which warrant hope. He will strive judiciously and skillfully to bring to bear all the potential mental agencies of which he may properly avail himself. He will throw on the scale of hopefulness all the weight to be derived from those doubts and difficulties which beset diagnosis and prognosis. He will make due allowance for the limitations of medical knowledge and his own deficiencies."—*Med. and Surg. Reporter.*

